# A statistical downscaling approach for generating high spatial resolution health risk maps: a case study of road noise and ischemic heart disease mortality in Melbourne, Australia

**DOI:** 10.1186/s12942-019-0184-x

**Published:** 2019-09-05

**Authors:** Ivan C. Hanigan, Timothy B. Chaston, Ben Hinze, Martine Dennekamp, Bin Jalaludin, Yohannes Kinfu, Geoffrey G. Morgan

**Affiliations:** 1 School of Public Health and University Centre for Rural Health, The University of Sydney, Sydney, Australia; 2 Ambient Maps, Rangeville, Australia; 3 Environment Protection Authority Victoria, Carlton, Australia; 4 School of Public Health and Preventive Medicine, Monash University, Clayton, Australia; 5 School of Public Health and Community Medicine, University of New South Wales, Sydney, Australia; 6 Centre for Research and Action in Public Health and Faculty of Health, University of Canberra, Canberra, Australia; 7 The Centre for Air Pollution, Energy and Health Research (CAR), Glebe, Australia; 8 Department of Health Metrics Sciences, University of Washington, Seattle, USA

**Keywords:** Environmental health risk assessment, Statistical downscaling, Exposure–response function, Noise, Mortality

## Abstract

**Introduction:**

Road traffic noise increases the risk of mortality from ischemic heart disease (IHD). Because noise is highly localized, high resolution maps of exposures and health outcomes are key to urban planning interventions that are informed by health risks. In Australia, publicly accessible IHD deaths data are only available at the coarse spatial aggregation level of local government area (LGA), in which about 130,000 people reside. Herein, we addressed this limitation of health data using statistical downscaling and generated environmental health risk maps for noise at the meshblock level (MB; ~ 90 people).

**Methods:**

We estimated noise exposures at the MB level using a model of road traffic noise in Melbourne, Australia, from 2011. As recommended by the World Health Organization, a non-linear exposure–response function for traffic noise and IHD was used to calculate odds ratios for noise related IHD in all MBs. Noise attributable risks of IHD death were then estimated by statistically downscaling LGA-level IHD rates to the MB level.

**Results:**

Noise levels of 80 dB were recorded in some MBs. From the given noise maps, approximately 5% of the population was exposed to traffic noise above the risk threshold of 55 dB. Maps of excess risk at the MB level identified areas in which noise levels and exposed populations are large. Attributable rates of IHD deaths due to noise were generally very low, but some were as high as 5–10 per 100,000, and in extremely noisy and populated MBs represented more than 8% excess risk of IHD death. We presented results as interactive maps of excess risk due to noise at the small neighbourhood scale.

**Conclusion:**

Our method accommodates low-resolution health data and could be used to inform urban planning and public health decision making for various environmental health concerns. Estimated noise related IHD deaths were relatively few in Melbourne in 2011, likely because road traffic is one of many noise sources and the current noise model underestimates exposures. Nonetheless, this novel computational framework could be used globally to generate maps of noise related health risks using scant health outcomes data.

## Background

### Noise and health literature review

Ambient environmental noise has been shown to impact cardiovascular disease (CVD) outcomes [1]. Studies from the environmental burden of disease in Europe (EBoDE) project show that noise pollution contributes significantly to rates of myocardial infarction (MI) for example [2]. Studies by the World Health Organization (WHO) (2011) also show that environmental noise contributes to CVD and high sleep disturbance (HSD) [3]. Currently the most reliable noise exposure estimates are those of road noise, but data have also been generated for air and rail traffic noise [3, 4]. The WHO and the EBoDE project intend to expand the scope to include health endpoints of annoyance, concentration and childhood educational outcomes. Further studies are also planned to assess the impacts of less prevalent/more intermittent sources of environmental noise, including construction, industry and cultural events.

Road traffic noise contributes 1.0–1.6 million disability adjusted life years (DALYs) annually in Western Europe [3] and has been associated with increased mortality and morbidity from hypertension [4], MI and stroke [1]. Epidemiological meta-analyses confirm exposure–response relationships between traffic noise and rates of coronary heart disease, with relative risks (RR) of 1.08 (95% CI 1.04–1.13; [4]) and 1.06 (95% CI 1.03–1.09; [5]) per 10-decibel (dB) increases in 24-h road traffic noise exposures across the 52–77-dB range. Similarly, in a study of diabetes and hypertension, an incident rate ratio of 1.11 (95% CI 1.05–1.18) was associated with 10-dB increases in domestic exposures to road traffic noise [6].

The physiological effects of noise are likely mediated by stress hormones such as catecholamine [7], which has been associated with increases in blood pressure and heart rate [8] and with changes in vascular endothelial function and arterial stiffness [9]. Noise related high sleep disturbance (HSD) similarly contributes to the risks of CVD and diabetes, likely via stress hormone related and other mechanisms. The effects of noise exposures on hypertension, IHD, stroke, diabetes, obesity and children’s blood pressure have been systematically reviewed [10]. Moreover, HSD has been mapped to areas of high traffic noise [11] and is considered an important outcome of environmental noise in cities, with significant financial implications for national health care systems. Geospatial maps of disease prevalence in cities, however, are limited to the spatial resolution of available data and are generally made available with substantial research costs. In the EBoDE studies, health outcomes data were collected in cohort and cross sectional surveys [12] and geospatial maps were generated at the street address level [13].

### Development of noise models

According to a 2009 review [14], the first health-relevant unit for road traffic noise (L_50_) was calculated in 1952 as the noise level in dB that is exceeded 50% of the time, and this metric was modeled with reference to traffic volumes (vehicles per hour) and distances from the center of the lane of traffic. Subsequent models included corrections for directly monitored noise levels and parameters for mean traffic speeds and percentages of heavy vehicles. To improve on the percentile metric, an equivalent sound density parameter L_eq_ was devised based on L_50_ weighted by noise levels that were exceeded for 10% and 90% of the time. After these formulae were adjusted to better model noise from heavy vehicles and reflections of noise from roadside buildings, the calculation of road traffic noise (CoRTN) procedure was developed and published in the UK in 1975. This model of hourly and 18-hourly exposures accommodates, in addition to the previous parameters, road gradients and surfaces, traffic flows, and noise from multiple lanes of traffic. In the 1988 update of this model, the effects of ground surfaces adjacent to major roads were included among corrections. Similar German (RLS90) and Italian (CNR) models were built on average hourly traffic flows of heavy and light vehicles and motorcycles, and in addition to accommodating average speeds, distances from the center of the lane and road types and dimensions, factors that influence sound propagation, such as vegetation, air absorption, reflection and refraction, were entered into the algorithms.

To inform land use, transport planning and design standards that affect community exposures to noise in Melbourne, Australia, the Environment Protection Authority (EPA) Victoria commissioned an assessment of noise levels from all sources throughout the metropolitan area [15]. The road traffic component of this study was performed using the Nordic Prediction Method 1996 (NPM96) with road traffic data from the Victorian Integrated Transport Model 2011 (VITM) [16]. Thus, we were equipped with a high-resolution traffic noise model for the entire city, but only annualized mortality counts for entire local government areas (LGA), of which there are 31 in this city.

### Downscaling health outcomes data

Whereas health-relevant spatial exposure data are increasingly available globally, they are rarely matched by health outcomes information at the same scale. In 2000, Künzli et al. estimated attributable cases of morbidity and mortality due to particulate matter of less than 10 μm (PM_10_) air pollution exposures at the 1-km^2^ level [17]. They devised statistical procedures to infer estimates from exposures and generated adjusted outcomes data. Fifteen years later, Apte et al. mapped PM_2.5_ related mortality across the entire globe at a resolution of 0.1° (longitude and latitude) [18]. They used age specific mortality data and population age structures for 21 international regions from the Institute for Health Metrics and Evaluation (Seattle, WA), and their algorithms produced aggregated estimates of the global burden of disease from PM_2.5_. We extend the previous work of Kunzli et al. and Apte et al. by developing a model for high resolution spatial estimates of health outcomes data using even smaller area residential population counts, and created an interactive mapping tool to enable policy and planning stakeholders to interrogate excess health risks due to road traffic noise. Because the effects of noise on health outcomes are localized, we accommodated the limitations of LGA level IHD mortality data by performing statistical downscaling computations using meshblock (MB; ~ 90 people per MB) population data from the Australian Bureau of Statistics.

The present computational framework is generalizable to other noise related health outcomes, such as IHD related hospital admissions and rates of HSD and annoyance and could be used to provide preliminary indications of noise associated health risks in data poor settings. In our application to Melbourne, this analytical framework identified locations for which IHD-related deaths may be mitigated by reducing road traffic noise exposures. This tool can be used to address public health concerns, such as those for which city planners and health policy makers are responsible.

## Methods

### Study region and period

The study region was the Melbourne greater metropolitan area in the state of Victoria, Australia (2011 population ~ 4 million). Data for the year 2011 were collected from the population census to correspond with the 2011 road traffic noise model. This is the most recent road noise data available. The study region was defined to incorporate all LGAs in which noise exposure-model data were estimated.

### Road traffic noise model

The EPA provided detailed maps of noise levels (measured as dB) that were developed from a project that used data from 45,000 road segments covering 7500 km of roads hosting over 2,000,000 buildings in a 10,000 km^2^ study region [15]. Noise maps of Melbourne were generated using the NPM96 with road traffic data from the VITM 2011. The algorithms of the NPM96 calculate A-weighted equivalent continuous sound pressure levels (L_Aeq_) from traffic flows of light and heavy vehicles, and consider speeds, distances to road center lines, heights of roads, heights, positions and thicknesses of barriers, types of ground surface and locations of exposed individuals. The model was verified by attended and unattended noise measurements at over 300 locations using ARL 315, ARL 316 and NTi XL2 environmental noise loggers during 2011. These noise loggers were used in accordance with the Australian Standard—AS 2702:1984. Noise levels were recorded as L_Aeq_ and A-weighted maximum noise levels (LA_max_) at 15- or 60-min intervals. A-weighting was applied to correct noise volumes relative to those perceived by the human ear, as the ear is less sensitive to very high and low audio frequencies.

Noise levels were modelled using SoundPLAN, which is an environmental noise modelling software suite from SoundPLAN GmbH. SoundPLAN was also used to develop detailed 3D models including ground contours, buildings, ground absorption, pavement surface types, noise barriers and various source data, such as percentages of traffic during day, evening and night-time periods that are predictive of road noise emissions. Predicted noise levels were verified against measured data, revealing 90% confidence between modelled and measured road traffic noise values to within ± 4–5 dB across day, evening and night periods. Using the verified noise model, noise levels were then predicted both at the façades of all floors for sensitive buildings in the study region, and as a noise grid covering the entire study area at a reference height of 1.8 m above ground level.

Noise estimates for the noise grid were aggregated to 10 × 10-m pixels, which were then averaged over MB areas using the boundaries from the ABS 2011 census. MBs contain about 90 people. Although noise levels vary substantially within MBs, these noise exposures are likely underestimates of those in homes that are close to noisy roads. Moreover, noise levels from road traffic were modelled using data from the VITM, which include traffic volumes for major arterial and feeder roads only. The limited coverage of small roads likely contributes to underestimates of noise exposures in MBs.

### Health data

LGA level IHD rates were obtained from the public mortality database of the Australian Institute of Health and Welfare: Mortality Over Regions and Time (MORT) books Local Government Area (LGA), 2011–2015 Table 2: Leading causes of death by sex, 2011–2015 [19]. To accommodate the limitations of LGA level IHD mortality data, prior to calculating attributable excess risks, we performed statistical downscaling computations using meshblock (MB; ~ 90 people per MB) population data from the Australian Bureau of Statistics. Specifically, mortality rates were downscaled to MBs using MB population data after adjusting for LGA-specific population weighted exposures (described below).

### Health impact function

To estimate noise-attributable excess risks of IHD deaths for all MBs in Melbourne, we calculated odds ratios (OR) of IHD in all MBs by inserting noise levels into the nonlinear polynomial that is recommended for relating traffic noise with IHD by the World Health Organization [11], as follows:

$$\begin{array}{l} {{\text{For}}\;\;L_{{{\text{day}},16{\text{h}}}} \ge 55\;{\text{dB,}}} \\ {} \\ \quad \quad \quad {OR\left( {L_{{{\text{day}},16{\text{h}}}} } \right) = 1.63 - 6.13 \times 10^{-4} \times (L_{{{\text{day}},16{\text{h}}}} )^{2} + \; 7.36 \times 10^{-6} \times (L_{{{\text{day}},16{\text{h}}}} )^{3} } \\ {} \\ {{\text{Else}}\;\;OR\left( {L_{{{\text{day}},16{\text{h}}}} } \right) = 1,} \\ \end{array}$$

where $L_{{{\text{day}},16{\text{h}}}}$ represents average noise levels between 0700 and 2300 h.

Excess risks are computed as follows:

$$ER_{i} = \left( {OR\left( {L_{{{\text{day}},16{\text{h}},i}} } \right) - 1} \right) \times P_{i} \times \widehat{{I_{k} }},$$

where $ER_{i}$ is the excess risk of deaths in MB_i_ and $L_{{{\text{day}},16{\text{h}},i}}$ represents the average exposure level in MB_i_. $OR\left( {L_{{{\text{day}},16{\text{h}},i}} } \right)$ is the odds ratio at that noise level and is estimated by inserting the MB estimated $L_{{{\text{day}},16{\text{h}}}}$ into the polynomial function above. $P_{i}$ is the population of MB_i_ and the baseline mortality incidence rate is represented by $\widehat{{I_{k} }}$. Our approach uses MB population numbers to calculate excess risks at the highest spatial resolution.

To estimate $\widehat{{I_{k} }}$ we used the regional average annual IHD mortality rate $I_{k}$ (for each region e.g. in $LGA_{k}$) and divided this by the population-weighted average OR of all MBs within region *k*, as follows:

$$\widehat{{I_{k} }} = \frac{{I_{k} }}{{\overline{{OR_{k} }} }},$$

where $\overline{{OR_{k} }}$ represents the population-weighted average OR within region k and is calculated using the following equation:

$$\overline{{OR_{k} }} = \frac{{\mathop \sum \nolimits_{i = 1}^{n} P_{i} \times OR\left( {L_{{{\text{day}},16{\text{h}},i}} } \right)}}{{\mathop \sum \nolimits_{i = 1}^{n} P_{i} }},$$

**Fig. 1:**
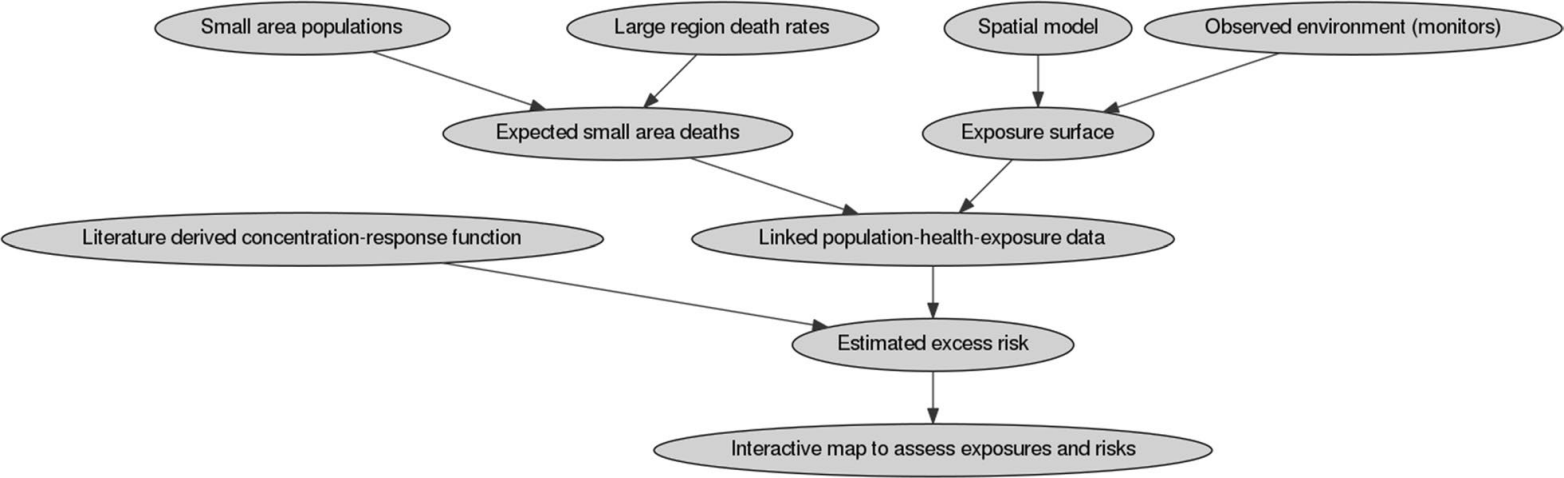
Flow chart of the working procedure for this study: (top left) small area ischemic heart disease (IHD) death rates are calculated from small area populations and large region IHD death rates using a downscaling approach; (top right) spatially weighted average noise exposures are estimated at the same spatial scale using a spatial model and directly monitored environmental data; (lower centre) spatially resolved population health and exposure data are entered into the concentration–response function to estimate excess risk and generate interactive maps of exposures and risks

These formulae represent the hypothetical underlying cause-specific mortality rate for region *k* and approximate the health outcomes that would be observed in a counterfactual unexposed population. Hence, multiplication of the resulting mortality rate by the population and the OR yields the attributable excess risk in person-years given the observed level of exposure. Figure 1 illustrates our risk assessment approach.

## Results

**Fig. 2:**
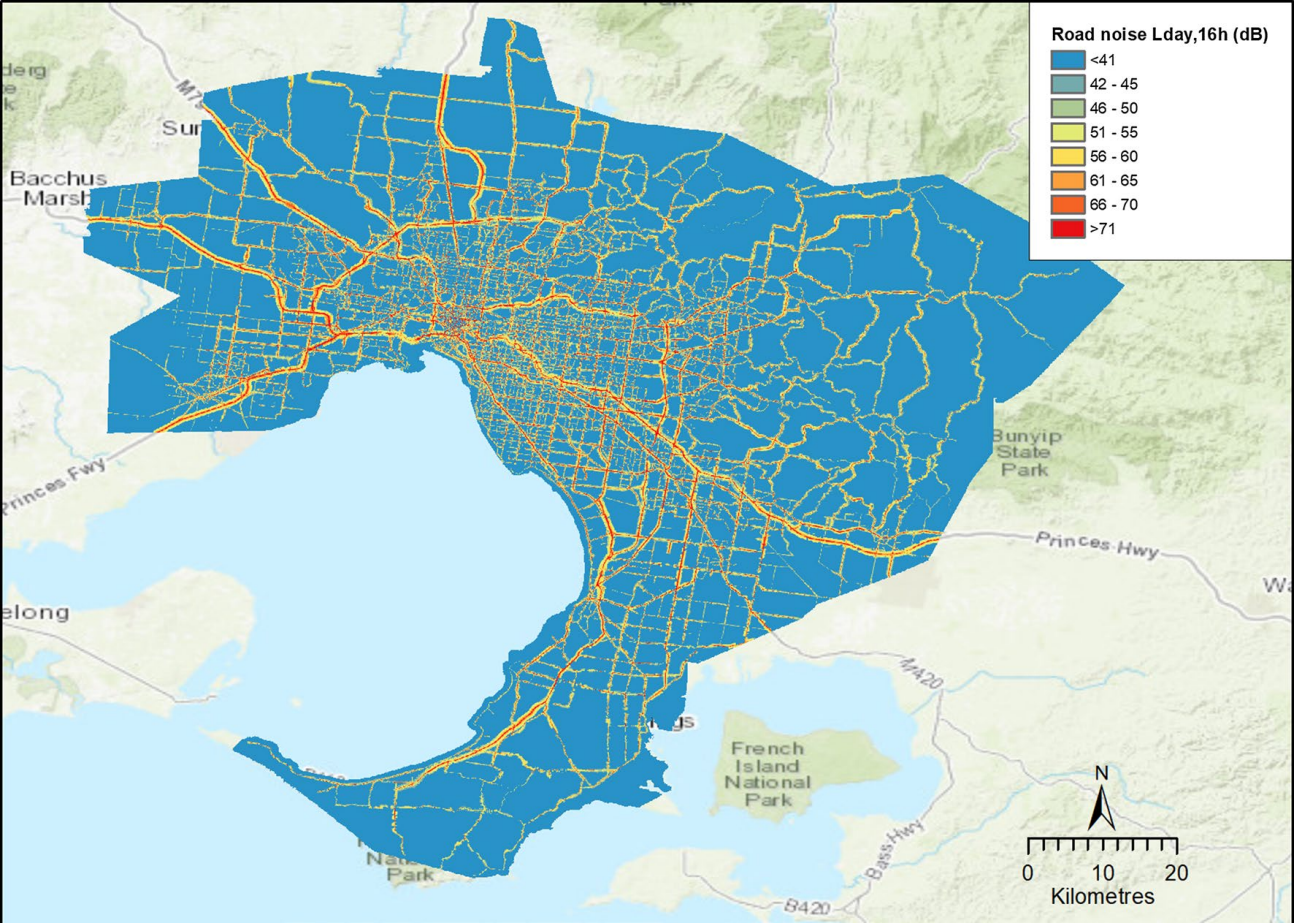
Greater Melbourne area and road noise model predictions (2011) at the level of 10 × 10-m pixels

We estimated exposures to average traffic noise over the 16 daytime hours 0700–2300 (labelled L_day,16h_, expressed in dB) within each MB. The average road traffic noise exposure for all MBs was less than 40 dB and the highest was 80 dB (in an unpopulated industrial MB). The study region and noise model are shown in Fig. 2 and descriptive statistics of MB noise exposure estimates and populations are listed in Table 1.

**Table 1:** Descriptive statistics of noise exposure levels in decibels (dB) and populations of meshblocks (N = 52,561)

	Number of mesh blocks	Mean	Median	SD	Min, max	10th %	25th %	75th %	90th %	IQR
Noise levels (dB) in all MBs	52,561	35.19	38.86	17.42	< 40, 80.43	2.62	25.58	47.81	54.6	22.23
Populations of inhabited MBs	43,541	90	87	48.44	3, 1516	36	62	114	142	52

*IQR* interquartile range

Percentages of the population exposed to noise categories are shown in Table 2. Only noise categories above 55 dB were considered relevant to IHD deaths according to WHO guidelines [11], and in Melbourne these were endured by about 5% of inhabitants during 2011.

**Table 2:** Percentages of the total population exposed to each noise category in the present noise model

Noise category (in dB)	Noise (population weighted dB)	Population	Percentage of population
Below 41	23.82	2,384,408	60.92
41–45	43.43	571,828	14.61
46–50	48.39	482,741	12.33
51–55	53.18	306,973	7.84
56–60	57.98	129,686	3.31
61–65	62.82	33,809	0.86
66–70	67.75	4529	0.12
71–75	71.69	121	0.00
76–80	NA	0	0.00
Total	NA	3,914,095	100.00

**Fig. 3:**
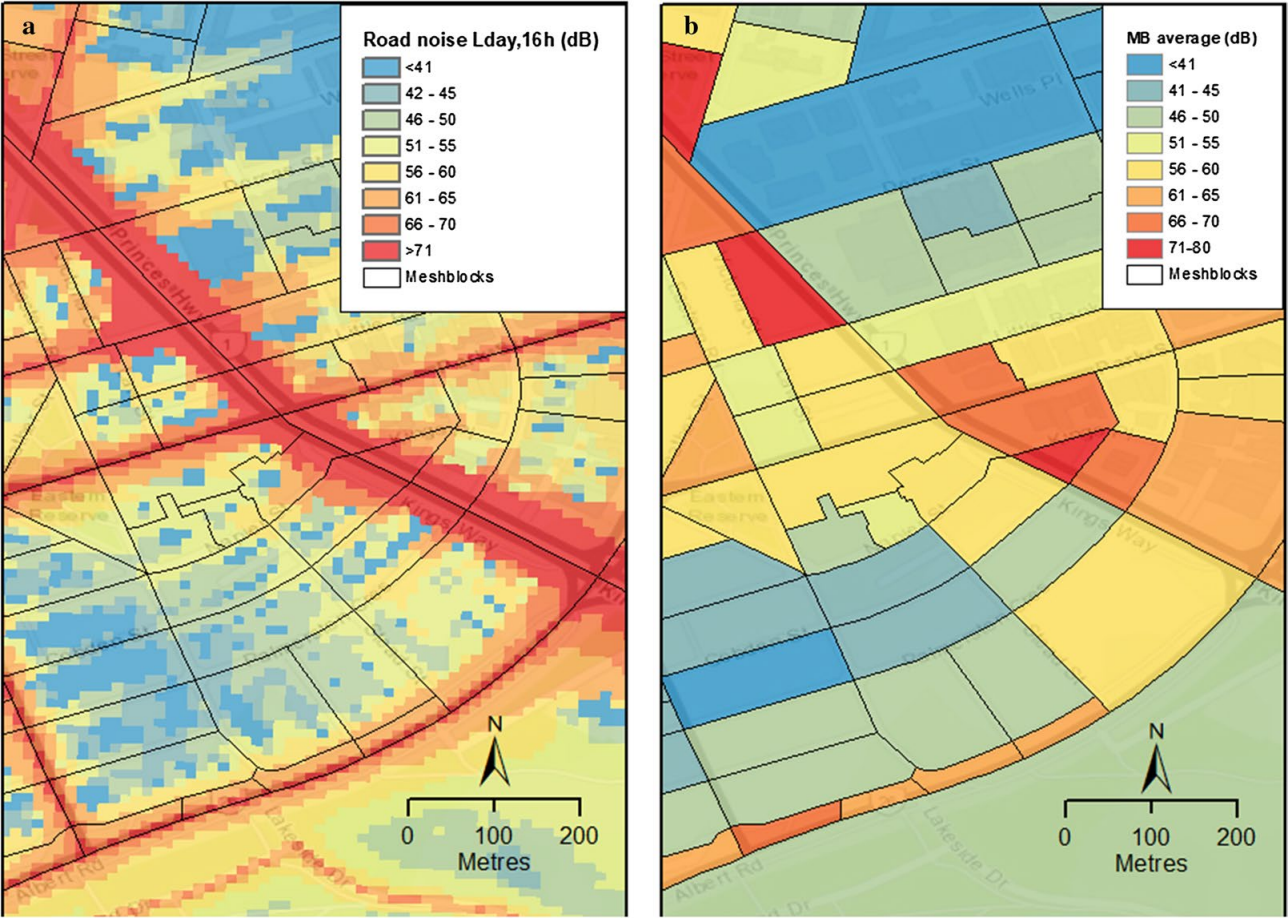
L_day,16h_ noise exposure at **a** 10 × 10-m pixels (left) and **b** meshblocks (MBs; right)

**Fig. 4:**
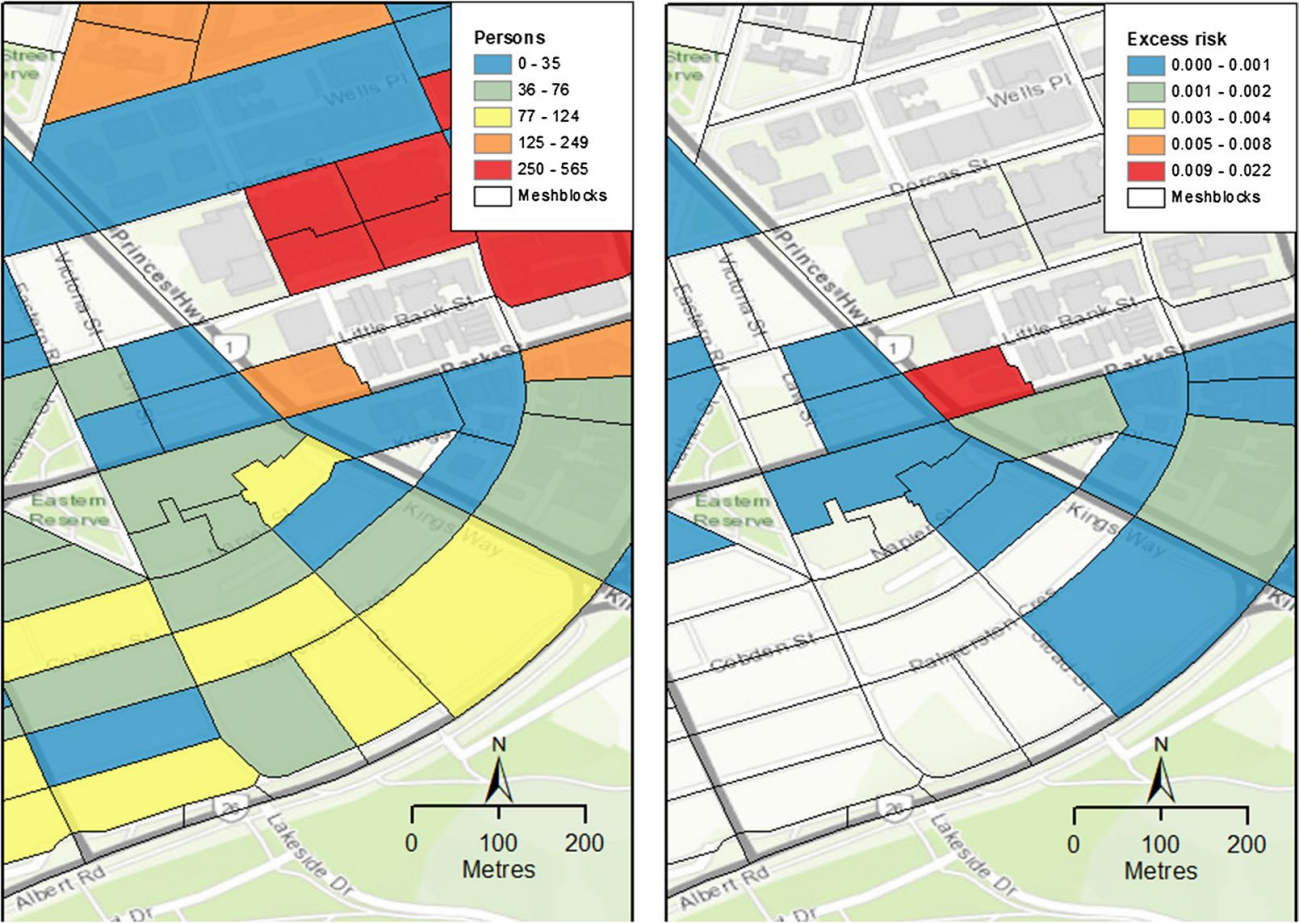
MB resident populations (left) and excess risks of IHD deaths due to road noise as a fraction of person-years (right)

The maps in Figs. 3 and 4 are of estimated noise levels and attributable excess risks in a selected neighbourhood for 2011. At this level, the maps show that the MBs of concern are those in which noise levels and exposed populations are large. In Fig. 3, distributions of noise (L_day,16h_) are shown in 10 × 10 m pixels (left panel) and as averages over MBs (right panel). Figure 4 shows resident populations of MBs on the left and estimated excess risks on the right.

Our estimated excess risks were typically very low across most MBs. Figure 4 shows a small area with high levels of road traffic noise and demonstrate differences between sides of busy roads. At this scale, some areas clearly have high noise levels but do not have appreciable excess health risks because they lack resident populations. In contrast, where high noise levels and high population numbers are coincident, attributable risks indicate the locations of potential health impacts of noise exposures.

Attributable rates of IHD deaths due to noise are an indicator of potential health impacts. In our calculations, these rates were generally very low for the majority of MBs, but were as high as 5–10 per 100,000 in some MBs. In the noisiest populated MBs, our computations suggest that greater than 8% of IHD excess death risk could have been avoided if noise exposure mitigation strategies had been employed.

As shown in Fig. 3, noise levels are very high (70 dB+) within 50 m of roads and quickly dissipate with distance from the source. Traffic noise levels were generally highest around major roads, in the central parts of the city and near industrial facilities. Because noise levels can vary substantially within MBs, averages of all pixels within MBs were used as estimates of noise exposures to residents. Noise associated health risks are logarithmically increased with sound intensity. Thus, even this level of spatial averaging considerably reduces estimates of noise-attributable IHD deaths.

**Fig. 5:**
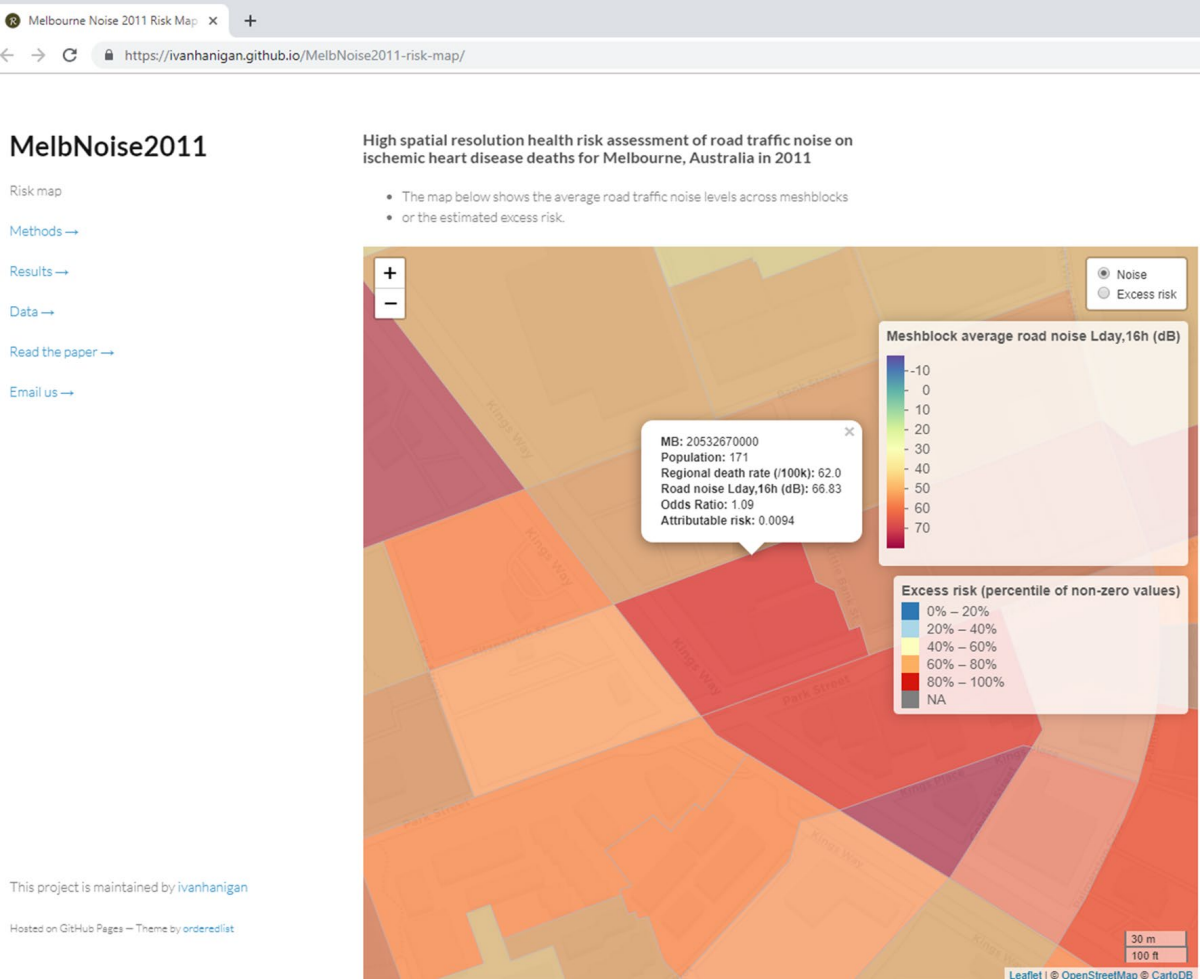
A small area of the interactive map for assessing exposures and risks

An interactive map was produced using the R package “leaflet” (http://rstudio.github.io/leaflet). In Fig. 5 we show an example of how to use the interactive mapping tool to zoom in and assess risks in MBs. This map is a sensitive tool for identifying potentially problematic areas, albeit with the limitations of low-resolution outcomes data and statistical downscaling assumptions. The interactive map is available as online Supporting Information at https://ivanhanigan.github.io/MelbNoise2011-risk-map/.

In Fig. 5, we show a labelled MB in the centre of the map with high noise levels and a population of 171. The regional LGA IHD death rate was 62 per 100,000, which could be expected in this MB under normal conditions. Yet the noise exposure of 66.8 dB equates to an OR of 1.09 and corresponds with an excess risk for IHD related death of 0.009 person-years in this MB.

## Discussion

We successfully demonstrated a new approach for creating high resolution maps of excess health risks due to environmental exposures when health outcome data are limited. We combined road traffic noise exposures and population health outcomes data using a statistical downscaling computational technique. Similar approaches have been used successfully by researchers to quantify excess risks of disease due to air pollution [17, 18]. We developed the method further to accommodate the absence of outcome data at a refined level of geography in Australia. We extended earlier research by applying the downscaling function that adjusts for population-weighted exposures across larger regions to estimate expected outcomes in very small regions of a population census. Although this is similar to the approach used by Apte et al., who used gridded raster population data prior to aggregating estimates to very large regions in their study of global health impacts from air pollution, our modifications to the approach allow robust estimation at very high spatial resolution. Our calculations are distinguished by population-weighted exposures that were calculated using enumerated population counts from the census. These provided greater precision and much smaller area resolution (MBs vs. 0.1° pixels of about 10 km at mid latitudes). We also developed a novel interactive online mapping tool that can be used to inform city planning and health policy. Finally, this is the first time that this statistical downscaling approach has been applied outside the field of air pollution. The present application of the method to the health impacts of noise demonstrates that this approach may be useful across a wide range of other environmental health risk factors.

Results from these analyses showed that during 2011, estimated traffic noise exposures (L_day,16h_) in MBs ranged from 20 to 80 dB (IQR = 22 dB) in the Melbourne metropolitan area. At these estimated noise levels, approximately 5% of the population would be exposed to road traffic noise above the IHD risk threshold of 55 dB [15]. Excess fractions of deaths attributable to traffic noise aggregated at the MB level were generally very low, but in extremely noisy and populated MBs, these represented greater than 8% of the total risk of IHD death.

Critical noise exposures of > 80 dB are more commonly experienced in occupational settings than in residential areas. Strong effects of workplace noise at these levels have been demonstrated in case control and cohort studies of cardiovascular outcomes [20–23]. The present road noise data set showed maximal mapped noise levels of 70–80 dB, even at main roads. Hence, whereas occupational noise is a demonstrated contributor to disease among people who work in industrial environments, the health effects of urban noise levels from road traffic may be more subtle, emerging only with long durations of exposure.

In a 2017 study, Dzhambov et al. assessed day-evening-night noise levels (L_den_) and night noise levels (L_night_) in 132 patients with hypertension living in the city of Plovdiv, Bulgaria [24]. Although their subject numbers were small, they collected and correlated clinical conditions with measurements of address- and room-level noise exposures. In their linear regression models, environmental noise was associated with increases in systolic blood pressure and decreases in estimated glomerular filtration rates, and these associations were stronger in patients with pre-existing CVD [24]. In a study of 6000 people in Belgrade, Serbia, Paunovic et al. indicated that 2.5% of MIs were attributable to road noise, and that men were more affected by road noise than women [25]. Exploiting similarly high resolution health data, Sorensen et al. demonstrated a MI incident rate ratio of 1.12 (95% CI 1.02–1.22) per 10-dB increase in road noise exposures in a cohort of 57,053 people, and reported a linear dose–response relationship [26]. Banerjee et al. also showed contributions of road noise to coronary heart disease risk, and whereas confounding effects of residence period, body mass index, and self-reported stress were identified, the authors deciphered the orientation of bedroom windows as a significant effect modifier [27].

Herein, the use of statistical downscaling assumptions allowed the production of high-resolution maps that can be used to zoom in on areas at most risk. Public health professionals, urban planners and development applicants could use these maps to identify areas in which the risks of noise are greatest and to assess developments in terms of their potential to contribute to disease. The present computations for the city of Melbourne may arrest public concerns relating to road traffic noise, at least in terms of IHD mortality, but the resulting maps specify locations for which road traffic noise should be considered during the planning of infrastructure and development projects.

The health impacts of urban noise exposures are increasingly considered in European and North American cities but are likely greater in poorer countries, where health outcomes are less likely to be assessed due to the lack of resources or will. We provide a low cost and robust approach for this problem in the form of a health risk mapping tool that requires only modelled estimates of noise based on more widely available traffic volume and population density data, which are comparatively inexpensive to gather. Application of this method to cities in which health outcomes data are limited will indicate locations at which health risks could be reduced with appropriate urban planning interventions. For example, city planners assessing applications for childcare centres or residential building developments could use this tool to evaluate whether the proposed sites have significant noise related health risks. Of greater impact in Melbourne and other cities globally, our method can be used to identify small residential areas in which the combination of high population numbers and noise levels currently represent significant health risks.

Our noise estimates were lower than those in noise models that have been generated for other cities, with previously reported average urban sound levels of about 58 dB [28, 29]. For example, in a study of birth weights, Smith et al. reported average A-weighted day and night noise levels of 58 and 53 dB, respectively, in the greater London (United Kingdom) area [28]. Whereas a mean L_den_ of 58 dB was reported for the comparatively small Belgian city of Ghent (population, 250,000; [29]); much higher than our average estimate of ~ 40 dB. Similarly, there was a much higher prevalence of census blocks with noise levels of > 80 dB in Ghent. We identified no such populated MBs in the city of Melbourne.

The present MB-level exposure data are likely underestimates of exposures at residential addresses due to the spatial averaging over MBs and omission of some smaller roads in the sound modelling and validation. Our averaging of noise levels across MBs would be the primary reason why the levels are lower in our study (i.e. sound pressure may be ~ 80 dB at the front of a building compared to 50 dB at the rear). These methods also rely on assumptions of homogeneous noise exposure levels within MBs, whereas noise is known to decrease exponentially with distance from the source. Yet smoothed average noise levels may be more representative of true exposures, because individuals move around their neighbourhoods with diurnal patterns and many are predominantly absent from their homes through the day. Such spatial smoothing could therefore merely introduce Berkson-type measurement errors rather than the more biased classical-type errors [30]. Regardless, this assumption is necessary to enable linkage with population data available from the census. We were also unable to distinguish between rooms and façades of buildings due to the averaging across MBs. Noise levels are known to vary widely within buildings. Hinze and EPA Victoria (2013) report maximum and average noise levels for the loudest façade and all façades, respectively, in Melbourne in 2011 [15]. Although the reported values are external, population exposures were also estimated in consideration of noise passing through the façade (windows). These data will be very useful in future studies, such as that reported by Banerjee et al., who showed that the orientation of bedroom windows significantly modifies the health risks associated with environmental noise [27]. Further validation with individual level data from studies using personal monitors is warranted to investigate this assumption. We expect that future noise models considering small roads, new roads and actual traffic speeds will show that disease-relevant road traffic noise exposures (i.e. noise levels > 55 dB) are widespread in the greater Melbourne metropolitan area.

Finally, it is worth considering the temporal alignment of our exposure and health outcomes data. We used noise data from 2011 with deaths data recorded over the period 2011–2015. Road traffic volumes will have changed and likely increased over that period, although where additional increases in traffic are possible, growth in volumes is typically less than 3% compounded yearly in the absence of new roads, new residential precincts or major road upgrades. The corresponding increase in noise levels over an eight-year period is approximately 1 dB. Therefore, we contend that the combination of these exposure and outcomes data appropriately represents the duration of exposure required to accumulate IHD risks in this population.

## Conclusions

The first spatial health risk maps of road traffic noise for IHD related death in Melbourne are presented in this paper. With scant health outcome data, we developed a computational mapping approach that can be used to estimate the risks associated with environmental noise and we present the results as an interactive online map tool to enable targeted interventions in areas of concern. This tool can be used when only coarse health data is available. Noise data can be modelled based on in situ measurements and maps of road noise sources. Our method along with new high-resolution spatial noise maps will make such environmental health risk assessments relatively easier to compute even when only low resolution health data are publicly available.

## Data Availability

The datasets generated during and/or analysed during this study are not publicly available due to licence conditions but are available from the corresponding author on reasonable request.
